# Barriers and facilitators to shared decision-making in hospitals from policy to practice: a systematic review

**DOI:** 10.1186/s13012-021-01142-y

**Published:** 2021-07-31

**Authors:** Alex Waddell, Alyse Lennox, Gerri Spassova, Peter Bragge

**Affiliations:** 1 Monash Sustainable Development Institute, Monash University, 8 Scenic Boulevard, Clayton Campus, Melbourne, VIC 3800 Australia; 2 Safer Care Victoria, 50 Lonsdale St, Melbourne, VIC 3000 Australia; 3 Department of Marketing, Monash Business School, Level 6, Building S, Caulfield Campus 26 Sir John Monash Drive, Caulfield East, VIC 3145 Australia

**Keywords:** Shared decision-making, Implementation, Hospital care, Barriers and facilitators, Theoretical Domains Framework

## Abstract

**Background:**

Involving patients in their healthcare using shared decision-making (SDM) is promoted through policy and research, yet its implementation in routine practice remains slow. Research into SDM has stemmed from primary and secondary care contexts, and research into the implementation of SDM in tertiary care settings has not been systematically reviewed. Furthermore, perspectives on SDM beyond those of patients and their treating clinicians may add insights into the implementation of SDM. This systematic review aimed to review literature exploring barriers and facilitators to implementing SDM in hospital settings from multiple stakeholder perspectives.

**Methods:**

The search strategy focused on peer-reviewed qualitative studies with the primary aim of identifying barriers and facilitators to implementing SDM in hospital (tertiary care) settings. Studies from the perspective of patients, clinicians, health service administrators, and decision makers, government policy makers, and other stakeholders (for example researchers) were eligible for inclusion. Reported qualitative results were mapped to the Theoretical Domains Framework (TDF) to identify behavioural barriers and facilitators to SDM.

**Results:**

Titles and abstracts of 8724 articles were screened and 520 were reviewed in full text. Fourteen articles met inclusion criteria. Most studies (n = 12) were conducted in the last four years; only four reported perspectives in addition to the patient-clinician dyad. In mapping results to the TDF, the dominant themes were Environmental Context and Resources, Social/Professional Role and Identity, Knowledge and Skills, and Beliefs about Capabilities. A wide range of barriers and facilitators across individual, organisational, and system levels were reported. Barriers specific to the hospital setting included noisy and busy ward environments and a lack of private spaces in which to conduct SDM conversations.

**Conclusions:**

SDM implementation research in hospital settings appears to be a young field. Future research should build on studies examining perspectives beyond the clinician-patient dyad and further consider the role of organisational- and system-level factors. Organisations wishing to implement SDM in hospital settings should also consider factors specific to tertiary care settings in addition to addressing their organisational and individual SDM needs.

**Trial Registration:**

The protocol for the review is registered on the Open Science Framework and can be found at https://osf.io/da645/, DOI 10.17605/OSF.IO/DA645.

**Supplementary Information:**

The online version contains supplementary material available at 10.1186/s13012-021-01142-y.

Contributions to the literature

- Research has shown involving patients in their healthcare using Shared Decision Making (SDM) in routine practice remains slow.
- The current study is the first qualitative systematic review of the barriers and facilitators to SDM implementation in hospital settings, and from the perspective of multiple stakeholders including patients, clinicians, health services administrators, health service decision makers, government policy makers, and researchers.
- The review findings add to previous SDM reviews by highlighting factors influencing SDM that are specific to tertiary care settings and reporting on the few studies that have incorporated perspectives of stakeholders beyond the patient and clinician.

## Introduction

Shared decision-making (SDM) is the process by which clinicians and patients (and/or their carers and families) come to a clinical decision regarding the next step to take in a patient’s health care [1, 2]. SDM involves a two-way exchange between the patient, who provides insight into their goals, values and preferences, and the clinician, who outlines the benefits, risks, and uncertainties of various care options based upon their experience and knowledge of the best available research evidence and recommendations [3]. SDM is underpinned by the practice of patient-centred care and the ethical belief that decisions should be made *with* patients instead of *for* them [4]. SDM is best suited to situations in which there is a clear need for a decision to be made, there is equipoise between care options, and it is feasible to engage in SDM conversations [5]. The SDM process can be modified to suit the context in which the decision is being made, and those involved may choose to take varying levels of responsibility for the decision [5, 6].

Including patients in decisions about their health care has long been seen as an ethical imperative [5]. Patient-centred care (PCC) integrates patient knowledge, while including patients’ wants, needs, and preferences in care decisions [7]. PCC and the inclusion of patients in decisions have been shown to increase patient engagement and satisfaction [8], decrease unwanted health service variation [9], and improve outcomes for disadvantaged patients [10]. Yet, despite increased focus from both policy and research, sharing healthcare decisions with patients is not yet part of routine clinical practice [11–13].

Systematic reviews of barriers and facilitators of SDM were conducted in 2008 [14], 2014 [15], and 2019 [16], focusing on clinicians, patients, and paediatric care respectively. The present review builds on this work in several substantive ways.

First, prior research has focused mostly on barriers and facilitators faced by patients and clinicians [13, 17–19]. SDM implementation, however, involves multiple stakeholders in healthcare systems. Stakeholders such as those working in health service administration or decision-making, government policy makers, and researchers may have insights not yet explored by research focusing on the patient-clinician dyad. A recent scoping review of organisational and systemic barriers and facilitators to SDM found a broad range that both drive and inhibit SDM implementation such as organisational culture and system-level guidelines and policies [13]. The present review contributes to the literature by exploring SDM barriers and facilitators from multiple stakeholder perspectives [20, 21].

Second, prior reviews have focused on SDM in primary and secondary care settings [22]. Primary care is usually the first point of healthcare contact and can include general practice, community health, or allied health services. Secondary care is defined as specialist care that patients are referred to by their primary care clinician and may include out-patient care or care in the community [23]. Primary and secondary care contexts (i.e. specific appointment times and time between appointments) are obvious settings to conduct SDM.

Compared to primary and secondary care, little is known about SDM in tertiary-care settings. Tertiary care involves medical or surgical treatment for patients, including emergency care, and usually over an extended period of time as an inpatient [23, 24]. There are more decisions to be made about patient’s healthcare while they are in hospital, providing increased opportunities to practice SDM. However, this presents challenges for SDM. Patients are likely to be more acutely sick and there may be increased time pressures to make decisions. Furthermore, staff workflows are also variable compared with primary and secondary settings, with changing shifts, busy ward environments, and more disruptions. The present research fills this gap by exploring SDM barriers and facilitators in tertiary care.

Lastly, the last decade has seen an exponential growth in SDM research [25]. A bibliometric analysis of this field reported year-on-year increases in the number of SDM publications, for example in 2009, n = 229 articles were published in this field, this rose fivefold to n = 1,199 as of 2018. As such, this review aims to build on previous reviews by synthesising new research within the exponentially growing field. Given the numerous stakeholders involved in SDM in hospital settings, it is important to consider the barriers and facilitators from multiple stakeholder perspectives [20, 21] and also consider the impact of hospital settings to optimise implementation [26]. Therefore, the aim of this systematic review was to synthesise evidence on the barriers and facilitators to the implementation of SDM interventions in tertiary care from the perspective of multiple stakeholders.

## Methods

### Design

The review approach was based on the Cochrane Qualitative and Implementation Methods Group and Handbook for Systematic Reviews [27], and reported in line with the PRISMA checklist [28]. The review protocol was pre-registered on the Open Science Framework (https://osf.io/da645/, DOI 10.17605/OSF.IO/DA645). Furthermore, experts were consulted prior to the review to ensure the relevance of the review for research and industry. These experts were especially interested in exploring the perspectives outside the patient-clinician dyad and how hospital settings may influence how SDM is implemented.

### Search strategy

The search strategy, designed in consultation with a speciality university-based librarian with subject matter expertise, aimed to include articles for which barriers and facilitators to implementing SDM in hospital settings were the primary focus and qualitatively reported. The MEDLINE, EMBASE, PsychINFO, CINAHL, Cochrane Library, and Scopus databases were searched for English language articles from January 2008 to July 2020. 2008 was chosen as the start year as research has already systematically reviewed patient and clinician barriers and facilitators prior to 2008 [14]. Reference lists of included studies were screened to identify additional eligible studies. Keywords used in the search string included “Shared Decision Making”, “Decision-Making”, “Patient Participation”, “Implementation”, “Attitudes” and “Beliefs” (example in Additional File 1).

### Study inclusion and exclusion criteria

The review used the SPIDER framework to frame inclusion and exclusion criteria (Table 1). The SPIDER framework is a modified version of PICO adapted for use with qualitative studies [29]. Where studies included both hospital inpatients and outpatients, only studies where more than half of participants were involved in decisions during their stay in hospital (i.e. while in emergency or as an inpatient) were included. Studies were excluded where barriers and facilitators to SDM were not the primary focus, for example those studies of the impact of SDM on outcomes. Studies were excluded if the majority of results were not qualitative as qualitative data is best suited to in-depth exploration of barriers and facilitators to SDM.

**Table 1:** Inclusion and exclusion criteria

	Included	Excluded
**Sample**	Patients aged 18 and over Healthcare providers Healthcare administrators Healthcare decision makers Government policy makers Other stakeholders (including researchers, not for profit organisations)	Patients aged under 18 years
**Phenomenon of interest**	SDM in hospital inpatient setting, in which the decision is made while the patient is an inpatient or in emergency	Non-SDM interventions Decisions made in primary or secondary care settings
**Design**	Primary studies where barriers and facilitators are qualitatively reported	Editorials Randomised control trials Quantitative studies Non-peer-reviewed studies Reviews (reviews were not included, but their reference lists were searched for additional primary studies)
**Evaluation**	Barriers and facilitators to implementing SDM in inpatient hospital settings where the decision is made while the patient is an inpatient, reported in the results section	Effectiveness of SDM interventions Impact of SDM interventions Preferences for decisions
**Research type**	Qualitative, mixed methods (qualitative only)	Quantitative, mixed methods (quantitative)

### Study selection

**Fig. 1:**
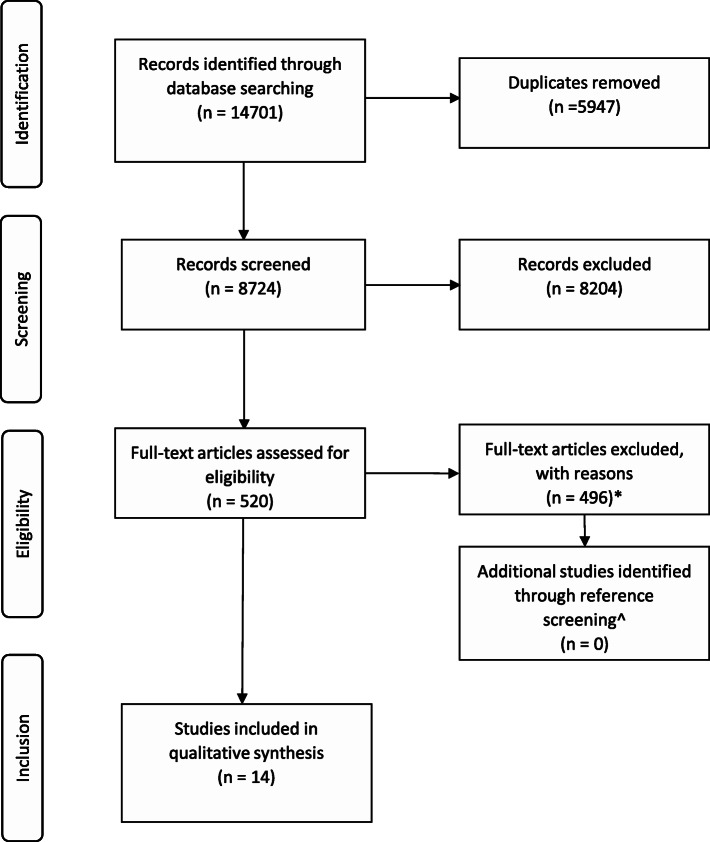
PRISMA diagram. *Of the 520 articles reviewed, n = 33 were excluded as the primary aim was not implementing SDM, n = 180 were excluded as the primary focus was not barriers and facilitators to implementing SDM, n = 68 were excluded as they did not qualitatively assess the barriers and facilitators to implementing SDM, n = 6 were excluded for using the wrong patient population, n = 98 were excluded as the context was not inpatient hospital, n = 11 duplicates were identified and excluded, n = 99 were the wrong study type. n = 1 study was excluded as the author did not respond to questions regarding methodology pertinent to study eligibility. ^n = 24 studies were included after full-text review, including n = 10 systematic reviews that were screened for additional studies, no additional studies were found.

The study selection process followed the PRISMA Checklist for reporting systematic reviews [28] (Fig. 1: PRISMA diagram; Additional File 1). Studies were uploaded to a purpose-built screening platform, Covidence [30]. After duplicates were removed, two reviewers (AW and AL) independently screened the title and abstracts of included articles. When reviewers disagreed, they discussed the articles until a conclusion was reached. When a conclusion could not be reached, a third reviewer (PB) adjudicated. The same process was used for full-text review. Reasons for excluding articles are reported in Fig. 1.

### Risk of bias (quality) assessment

The methodological quality of included studies was assessed based on the Critical Appraisal Skills Programme (CASP) quality assessment tool for qualitative studies [31, 32] (Additional File 3). The CASP tool asks researchers to assess the usefulness of the articles for inclusion and to identify any methodological issues. Consistent with previously published approaches [33], the tool was modified to use “can’t tell” when there was not enough information to make a judgement and included a “somewhat” option. CASP findings were used to assess the confidence of the review findings using the GRADE-CERQual “Confidence in the Evidence from Reviews of Qualitative Research” (CERQual) tool [34]. CERQual is a novel approach to systematically assessing confidence in review findings using methodological limitations, coherence, adequacy, and relevance [34–39]. These components were individually assessed for each of the review findings, and marked as having either “no or very minor”, “minor”, “moderate”, or “serious concerns”. Overall assessment using the components was then determined as “high”, “moderate”, or “low confidence”.

### Data synthesis and presentation

Following study selection, one reviewer (AW) extracted the following data from included studies: article reference, country of origin, primary and secondary study objectives, use of conceptual or theoretical framework, study design, participant characteristics/role, target adopters, description of the innovation/implementation strategy (if used), description of the practice environment, outcomes and when measured (barriers and/or facilitators), and limitations. A second reviewer (AL) completed over 10% of the data extraction and this was compared.

Data analysis and synthesis drew upon direct quotes from study participants where possible; where direct quotes were not provided, the author’s interpretation was used. Analysis involved two phases.

In the first phase, a “Best Fit Framework Synthesis” (BFFS) [40–42] was used. The BFFS allows for synthesis to be based on a previous published model. Therefore, previously published taxonomies of barriers and facilitators to SDM for patients and clinicians [14, 15] were used as a basis for data synthesis. These were amended through inductive coding to include barriers and facilitators for government policy makers and health services.

In the second phase, the codes identified in phase one were coded to the Theoretical Domains Framework (TDF) [43]. The TDF [43] was identified as the most appropriate analysis framework as this enabled affective, cognitive, social, and environmental factors influencing behaviour to be explored [26]. Mapping barriers and facilitators to the TDF for multiple stakeholders can highlight areas in which factors align. This may allow future implementation programmes to address multiple factors for multiple stakeholders.

## Results

### Results of the search

Of 14701 records, 8724 were screened for inclusion based on title and abstract (Fig. 1). Of these, 520 were further screened based on the full text. 14 articles were deemed to meet all inclusion and exclusion criteria [44–57]. A review of reference lists of relevant systematic reviews did not identify any additional studies for inclusion.

### Study characteristics

**Fig. 2:**
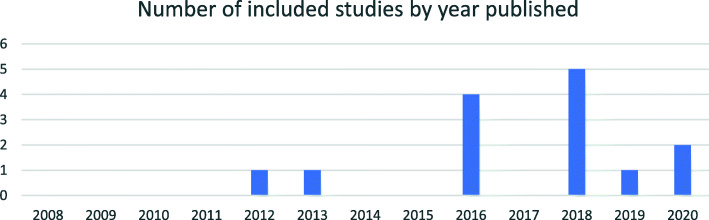
Included published studies by year

Included articles were published between 2012 and 2020, with the majority of articles (n = 12) published in 2016 to 2020 (Fig. 2).

Included articles used qualitative study designs, with the majority using interviews [44, 46–48, 50–52, 55–57], followed by focus groups [44, 46, 51, 54], observation [49, 53], and conference breakout session [45].

Seven countries were represented across the included articles including the USA [45, 47, 48, 55–57], Canada [44, 49, 57], Germany [53, 54], The Netherlands [46], Australia [50], UK [51], and France [57] (Additional File 2).

Of the included articles, the majority focused on emergency department settings [45, 47, 48, 55, 56] and acute mental health settings [50, 51, 54], with other settings including cardiology [52, 57], oncology [53], stroke rehabilitation [44], and acute monitoring [49]. There were 11 authors for 14 articles, with four separate articles by Schoenfeld included [47, 48, 55, 56]. These articles also represented the majority of articles included regarding SDM in the emergency department.

A wide range of barriers and facilitators across individual, organisational, and system levels were reported with many overlapping across the TDF. Reported barriers and facilitators to SDM in inpatient settings ranged across all 14 domains of the TDF [43] (Table 2), with the majority relating to clinician-related factors, followed by patient-related factors, organisation-related factors, system-related factors, and finally other stakeholder-related factors. Overall, the dominant themes (themes cited most frequently) occurring across clinician-, patient-, organisation-, and system-related factors were “Skills” (such as clinicians’ lack of formal training to do SDM); “Knowledge” (such as patients’ limited understanding of risk); “Environmental Context and Resources” (such as noisy and busy ward environments); “Social/Professional Role and Identity” (such as clinicians’ perceived role as decision maker); and “Beliefs about Capabilities” (such as patients’ belief that they should be included in decisions about their care) (Additional File 4). The majority of included studies (n = 13) explored the perspective of clinicians; a smaller number explored patient perspectives (n = 6). Of the Health Care Provider (clinicians) perspectives included, the majority were medical doctors [44–46, 48, 50–57], followed by nurses [49–51, 57] and other allied health professionals [50, 51, 57]. Only four studies included the perspectives of stakeholders other than the patient-clinician dyad, such as health service programme administrators [44–46, 57], health service decision makers [45, 46, 57], government policy makers [46, 57], and other stakeholders (such as researchers) [45, 46, 57].

**Table 2:** Barriers and facilitators to implementing SDM data mapped to the Theoretical Domains Framework (Cane et al., 2012) from multiple perspectives

Stakeholder group	Clinician-related factors	Patient-related factors	Health service administrators (HSA), Decision makers (HSDM), Government policy makers (GPM), and other stakeholder-related factors	Organisational-level factors	System-level factors
1. **Knowledge (An awareness of the existence of something)**					
Barriers	- Not knowing what SDM is [57] - Incorrect definition of SDM [46, 52, 55, 57] - Assumes patient understands information shared [53] - Does not know true risk of options [56]	- Limited or no knowledge of disease or options [46, 47, 50, 54] - Limited understanding of risk [44, 45, 50, 52, 53, 56] - Not provided adequate information for decision [53, 55] - Provided biased information [50, 55] - Not understanding jargon used by clinicians [51, 53] - Not knowing own patient history (i.e. previous drug treatment) [54] - Not having knowledge of language to describe their experience of illness [51]			- Lack of guidelines that include SDM [46]
Facilitators	- Understanding of SDM and what it entails [47, 49] - Understanding of risks and benefits for treatment options [47–49]	- Well informed about the disease and treatment options prior to the SDM conversation [45, 47, 50, 52–54, 57] - Patient is able to understand consequences and risks of alternatives [45, 47, 50–53] - Knowledge of previous treatments for condition (i.e. which drugs they had been treated with previously) [54]		- Use posters/reminders to create awareness of SDM implementation programme [44, 49] - Tailored information services for patients [46]	- Support cross-site learning through regular meetings [44, 46] - Pool information from separate SDM initiatives to speed knowledge translation [46] - Promote awareness of the benefits of SDM through research [55] - Promote patient awareness of SDM through national campaign [46]
2. **Skills (An ability or proficiency acquired through practice)**					
Barriers	- Lack of training in SDM [44, 48–51, 55, 56] - Lack of communication skills [51, 55] - Lack of skills to train junior doctors in SDM [48, 56] - Overreliance on clinical algorithms for determining treatment decisions [48, 56]	- Decision is left to the patient [53] - Informational capacity to make informed decisions (barrier and facilitator) [45, 47, 50, 52, 54, 56]		- Senior clinicians are expected to teach junior doctors how to do SDM without having training themselves [48, 56]	- Lack of training to do SDM [46, 48, 50, 51, 56]
Facilitators	- Communication skills, i.e. ability to explain risks and benefits of treatment option [45–48, 50–53, 56, 57] - Formal training in SDM [44, 48–51, 55, 56] - Trust in one’s own clinical skills and ability [47, 55–57] - Awareness of one’s own limitations as a clinician [55, 57] - Has been given education in communication with patients [48, 50, 51, 57] - Uses evidence-based data to inform treatment options [45] - Experience increases clinical skill and confidence [56] - Providing tailored information to patients based on their informational needs [50]	- Informational capacity to make informed decisions (barrier and facilitator) [45, 47, 50–52, 54, 56] - Ability to speak up for own preferences due to prior experience in health setting (i.e. as nurse or long-term patient) [45, 47, 54]		- Require full team interdisciplinary training to ensure language is the same across disciplines when implementing SDM [44, 50] - Provide training on use of Patient Decision Aids [44, 46, 57] - Opportunity to practice SDM with senior clinicians [48]	- SDM is part of medical student’s education [46, 48] - Including patients in SDM education [46] - Support cross-site learning through regular meetings [46]
3. **Social/professional role and identity (A coherent set of behaviours and displayed personal qualities of an individual in a social or work setting)**					
Barriers	- Clinicians belief that their role is to make decisions and convince patients [50, 52, 56] - Not wanting to seem indecisive [48, 56] - Belief that colleagues do not want to do SDM [55, 57] - Patient has no or limited ongoing primary care [50, 56]	- Belief that clinician’s role is to make decisions [47, 57] - Belief that nurses should not be involved in SDM [49] - Not wanting to be labelled “difficult” [45, 46, 53] - Perceived unacceptability of asking clinician questions [45] - Social stigma of having and seeking treatment for mental illness [50]			
Facilitators	- Clinician sees role as educator of patients [46, 49, 50, 52, 53] - Clinician sees role as collaborator with patient [46, 47, 49, 50, 53–55] - Asks for patient’s preferred role in SDM [45, 51, 53] - Interprofessional collaboration—clear communication [45, 46, 49–51, 55] - Nurse is involved in SDM [49, 54]	- Has positive/trusting relationship with clinician [47, 50, 51, 54, 55] - Belief that it is their role to be involved in decision-making with clinician (i.e. asks questions) [46, 47, 53] - Feeling more comfortable speaking with allied health (i.e. pharmacist) [50]	HSA, HSDM-related factors - Manages implementation through actively anticipating personnel/budget shifts [46] - Sees duty in aiding implementation of SDM through knowing appropriate education is being provided to clinicians and patients [57] Other stakeholder-related factors - Engage new policy makers in SDM [46]	- Engage new clinicians/patients in SDM [46]	- Include SDM in professional role descriptions for clinicians [46] - Showcase innovators of SDM [46] - Show patients their role in SDM through national campaigns [46]
4. **Beliefs about capabilities (Acceptance of the truth, reality, or validity about an ability, talent, or facility that a person can put to constructive use)**					
Barriers	- Clinician belief that patient does not want to “do” SDM [45, 50, 54, 56, 57] - Clinician belief that the patient will make the wrong choice [55, 56] - SDM is too much effort [56, 57]	- Patient belief that patients should not disagree with the clinician [45, 47, 56, 57]		- Belief that change is too difficult, takes too long, too many resources needed [46, 57]	- Change is too difficult, takes too long, too many resources needed [46, 57]
Facilitators	- Belief that patients should be involved in decisions about their own care [47, 50, 52, 55] - Risk is part of medicine [52, 55] - Acknowledges own biases that may interfere with decision-making [46]	- Belief that patients should be involved in decisions about their own care [45, 47, 53, 54]			
5. **Optimism (The confidence that things will happen for the best or that desired goals will be attained)**					
Barriers	- Belief that colleagues will not want to do SDM [55, 57] - Belief that SDM carries increased risk of litigation [45, 56]	- Lack of confidence in their clinician and/or outcome [56]			Change is too difficult, takes too long, too many resources needed [46, 57]
Facilitators		- Having trust and patience in the treatment decision and expecting a good outcome [51, 54]			
6. **Beliefs about consequences (Acceptance of the truth, reality, or validity about outcomes of a behaviour in a given situation)**					
Barriers	- Fear of a negative outcome [45, 47, 49, 55, 56] - Disease is too acute for SDM [47, 52, 56, 57] - Lack of applicability of the clinical situation [45, 50]	- Fear of negative consequences of the eventual decision [47]			
Facilitators	- SDM reduces healthcare utilisation [55, 56] - SDM aids decision-making [52, 55, 57] - SDM improves relationships between clinicians and patients [46, 50, 55, 57] - SDM increases patient satisfaction and sense of control [55, 56] - SDM eases the burden on clinicians (i.e. makes work easier) [57] - Including patients in SDM reduces the likelihood of litigation [55]			- Patient decision aids can help stimulate SDM conversations in busy environments [46]	
7. **Reinforcement (Increasing the probability of a response by arranging a dependent relationship, or contingency, between the response and a given stimulus)**					
Barriers	- Potential for litigation [45, 55] - Not motivated by patient satisfaction metrics [55, 56] - Not motivated by the potential benefits of practising SDM [55] - Not motivated by reduced healthcare utilisation [55]			- Quality assurance tools do not promote SDM [46]	- Risk of litigation for clinical mistakes [55] - Lack of reward for doing SDM [46]
Facilitators	- Motivated by patient satisfaction [55] - Positive experiences engaging patients in SDM [56]				- Changing legislation to reduce clinician’s risk of being sued for mistakes [55] - Use financial incentives to reimburse time spent doing SDM [46] - Include SDM in professional audits [46]
8. **Intentions (A conscious decision to perform a behaviour or a resolve to act in a certain way)**					
Barriers	- Deciding the treatment plan before speaking to the patient [47, 50–53, 55] - Intending to “sell” the patient on the chosen treatment option [47, 50, 52, 55] - Compliance as motivator [47, 48, 55, 56] - Intentionally not engaging in SDM when junior doctor is the first to see the patient [48, 56] - Leaving the patient to make the decision [53]	- Non-adherence with treatment plan [54, 56]		- Teams deciding together on the best course of action without input from the patient [53] - Not replacing personnel in charge of SDM programme [57] - Not providing support coverage for nurses to attend SDM training sessions [44]	
Facilitators	- Intentionally asking patient preferences [45, 50] - Seeking to understand and alleviate patients concerns [45] - Seeking to understand individual needs of the patient [45, 53] - Wanting to reduce harms of unnecessary and potentially harmful testing (i.e. CT scan) [55]	- Being open and honest with clinician about feelings, fears and preferences [50, 54] - Asking questions and providing feedback about symptoms/treatment [54] - Being open and honest in discussions around treatment [54] - Deciding to cooperate with treatment plan [54]	- Facilitate connections between multiple SDM implementation sites i.e. through community of practice [46]		
9. **Goals (Mental representations of outcomes or end states that an individual wants to achieve)**					
Barriers		- Patient lack of engagement or ambition [50, 54]			
Facilitators				- Seeking to implement SDM using Patient Decision Aids [49, 57]	- Bringing individual programmes together with the goal of sharing learnings in order to facilitate knowledge creation [46]
10. **Memory, attention, and decision processes (The ability to retain information, focus selectively on aspects of the environment, and choose between two or more alternatives)**					
Barriers	- Interruptions make it difficult to concentrate on engaging in SDM [48, 53, 56] - Competing priorities, i.e. highly acute patients/time make it easier to order more tests rather than engage in SDM [48] - Reliance on algorithms to make clinical decisions [48, 52, 55]	- Significant decision—difficulty being objective [45, 47, 50, 57]		Fear that implementing SDM will interrupt workflows [46, 57]	
Facilitators	- SDM draws attention to clinician’s own biases [46] - Significant decision requires additional attention and patient preference [45]	- Increased attention recognising it is a significant decision [45]			
11. **Environmental context and resources (Any circumstance of a person’s situation or environment that discourages or encourages the development of skills and abilities, independence, social competence, and adaptive behaviour)**					
Barriers	- Condition is too acute for SDM [47, 52, 56, 57] - Lack of time to engage in SDM [44, 45, 47–50, 53, 56, 57] - Noisy or busy ward environment [47, 48, 51, 53, 56] - Lack of private space to conduct SDM conversations [47, 50, 51, 56] - Patients often placed in hallways (not feasible for SDM conversation) [47, 48, 51, 56] - Presence of family/carers [49, 51, 56] - Clinician characteristics [52] - Interprofessional collaboration allows for more time for the decision to be made [50]	- Patients characteristics such as lower socioeconomic status, multiple comorbidities, lack of clinician language, past negative health experiences [45–51, 55, 56] - Lack of primary care physician to follow up with treatment decisions [50, 56] - Not having sufficient time for decision-making [53]	HSA, HSDM-related factors - Implementing SDM will take too much time, or too many resources [57] Making changes within the healthcare system is too difficult [57]	- Noisy or busy ward environment [47, 48, 50, 51, 53, 56] - Lack of private space to conduct SDM conversations [47, 51, 56] - Patients placed in hallways (not feasible for SDM conversation) [47, 48, 51, 56] - Not enough clinicians [47] - Waiting time to see clinician [47, 56] [11, 12] - Resources not available to use Patient Decision Aids [57] - No process for contacting primary care physicians on discharge [56]	- Inadequate funding of SDM [46] - Lack of agreed national plan for SDM [46] - Lack of clinical guidelines supporting SDM/fragmented availability of guidelines [44, 46, 55, 57] - Lack of decision-making materials (i.e. patient decision aids) [46, 57] - Part of policy, but not enforced i.e. through quality measures [46, 55]
Facilitators	- Clinical equipoise of treatment decision [52] - Low acuity, meaning more time for SDM discussion [52] - Including the family in SDM [45, 49, 50] - Including the patient in decision-making as soon as possible (i.e. when first arriving on the ward) [51] - Using communication tools that explain risk [56] - Having minimal people involved in SDM conversation, as too many people can bring in different opinions [56]	- Patients characteristics such as higher socioeconomic status, education, health literacy [45–51, 55, 56] - Presence of a carer/family [45, 47, 49–51, 53] - Carer/family providing translation support [54] - Using question prompt lists [46]	HSA, HSDM-related factors - Past negative experience with SDM [54] Other stakeholder-related factors - Monitor SDM implementation [46]	- Any SDM intervention is supported by evidence-based literature [46, 49] - Using a standardised channel (i.e. form) for sharing information across teams [44, 49] - Ensure forms can be modified in line with needs of the team [44] - Private spaces to conduct SDM [50]	- Change guidelines to promote use of SDM in clinical practice [44, 46, 55, 57] - Create locally based, context-specific SDM implementation evidence [44, 46] - Research into the specific benefits of SDM tools [55] - Allow patient access to medical records [46]
12. **Social influences (Those interpersonal processes that can cause individuals to change their thoughts, feelings, or behaviours)**					
Barriers	- Senior clinicians not engaging in SDM [46, 48, 57] - Other clinicians not engaging in SDM [55] - Inconsistent messaging between interprofessional team members [50, 51]	- Perceived power imbalance between the clinician and patient [54] - Family pressure to choose a particular treatment option - Cultural beliefs [45, 49, 56]	HSA, HSDM-related factors - Not having a site champion/leaders to endorse implementation of SDM [46, 48, 57]	- Lack of team support for clinician to do SDM [57] - Lack of organisational role models promoting SDM [46, 48, 57]	- Lack of support from policy makers [57]
Facilitators	- Senior clinicians engaging in SDM [46, 48, 57] - Consistent messaging between interprofessional team members [45, 50, 51]			- Culture of the organisation supports SDM [46, 48, 55–57] - Leadership engages in SDM [44, 46, 48, 57] - Conduct regular SDM implementation team meetings [44] - Establish site champions for SDM [44, 57]	
13. **Emotion (A complex reaction pattern, involving experiential, behavioural, and physiological elements, by which the individual attempts to deal with a personally significant matter or event)**					
Barriers	- Fear of “missing something” [45, 56, 57]	- Fear of uncertain or negative outcomes [45, 47, 49, 55, 56] - Patient being perceived by the clinician as being “rude” or “aggressive” or not open to SDM [47, 51, 54] - Fear of being labelled difficult [46, 53, 54] - Feeling like clinicians are not listening to concerns [51] - Feeling stressed due to busy or noisy ward environment [51] - Family members are emotional and stressed [51] - Feeling powerless during involuntary admission [54] - Reduced desire to engage in active decision-making (due to illness) [54]			
Facilitators		- Feeling listened to [51, 54] - Patient being calm and respectful [54]			
14. **Behavioural regulation (Anything aimed at managing or changing objectively observed or measured actions)**					
Barriers		- Not following treatment plans [47, 54]			
Facilitators	- Clinician taking a full medical history to encourage patient preferences [54]	- Following treatment plan [54] - Asking to be involved in decision-making [47, 54] - Asking questions in the consultation [47, 54, 55] - Opposing treatment recommendations [47, 54, 57] - Researching own illness/treatment [54] - Giving feedback on treatment experience [54]		- Change clinician habits through changing care processes to include patient preferences [46] - Create mandatory reporting of SDM implementation programmes [44] - Posters around ward to remind nurses of SDM implementation [49] - Engaging all patients in decision-making as soon as possible when they enter the ward [51]	

Four studies reported on barriers and facilitators in the context of implementing specific SDM programmes. These encompassed implementing SDM using a knowledge translation approach [44]; utilising the “three talk collaborative deliberation model” of SDM [53]; and harnessing patient decision aids [49, 57].

### Study quality assessment and overall confidence in the evidence

Overall, study quality was high with the majority of studies clearly stating the aims of the research and using appropriate research design, recruitment, and data collection to answer the aims. Furthermore, ethical issues were taken into consideration, and data analysis and statement of findings were clear. Some studies did not adequately report on the relationship between researcher and participants [45, 49, 51, 53, 54]. Two studies were of low quality [45, 49], as they did not adequately report their research design or data collection. Additionally, their data analysis and findings were not clear as they did not attribute findings to participants or make clear how conclusions were drawn from the data.

Table 3, Table 4, Table 5, and Table 6 present findings, including confidence in the evidence based on GRADE-CERQual for clinicians, patients, and other stakeholders respectively. Overall there were minor concerns with methodological quality as assessed by CASP. There were minor concerns with coherence with some studies contributing to findings based on authors interpretation and thematic analysis without the use of quotes. Adequacy and relevance tended to be of no or very minor concern; however, findings including studies by Schoenfeld et al. [48, 55, 56] were of moderate or low confidence as these three studies were based on the same interviews of n = 15 emergency department physicians.

### Dominant themes

Table 3 shows the dominant reported themes for clinician-, patient-, organisation-, and system-related factors mapped to the TDF. Themes cited four or more times were considered dominant themes. Given only three of the 14 studies included stakeholders outside the patient-clinician dyad, stakeholder-related factors are presented separately. Of the key themes reported, the dominant themes included “Skills”, “Knowledge”, “Environmental Context and Resources”, “Social/Professional Role and Identity”, and “Beliefs about Capabilities” (Additional File 4). Dominant themes specific to Clinician-, Patient-, Organisation- and System-related factors are reported in Table 4, Table 5, and Table 6 respectively.

### Skills and knowledge

“Skills” and “Knowledge” were commonly reported together as factors influencing the use (or non-use) of SDM. Review findings were of either moderate or high confidence with the majority being high confidence.

#### Clinicians

Clinician skills influence the practice (or non-practice) of SDM. A number of clinicians report a lack of formal training in SDM [44, 48, 49, 51, 56] and communication [51, 55] meaning they are unsure if they are doing SDM correctly, or under which situations it would be best suited. Clinicians recognise the importance of communication skills, including how to communicate effectively with patients in order to explain risks and benefits and elicit preferences [45–48, 50–53, 56, 57]. Some clinicians feel they or others would benefit from specific training in communication [48, 50, 51, 57] in order to better facilitate SDM conversations. Further to this, trust in one’s own clinical ability is seen as a facilitator of SDM [47, 55–57], with clinicians’ past experience allowing them increased clinical skills and confidence [56] and awareness of their own limitations [47, 48, 55–57]. Not knowing what SDM is or what it entails is a barrier for clinicians; however, this is not reported by clinicians themselves, rather the included studies report instances of clinician participants using incorrect working definitions [46, 52, 55, 57].

#### Patients

Patients who are well informed prior to having SDM conversations, either by gathering information themselves or being given suitable information, report feeling better able to participate in SDM conversations and form opinions [45, 47, 50–54, 57]. This is especially true for patients who understand the risks and benefits of the different treatment options [45, 47, 51–53]. However, the way in which clinicians present information (for example not providing adequate information [53, 55] or purposely providing biased information [50, 55] may prevent patients from being well informed.

Conversely, lack of knowledge for patients is a barrier to engaging in SDM. Patients report not being provided adequate information to understand their options. This is also a barrier for clinicians who find it difficult to have SDM conversations with patients who have little knowledge of their disease [44, 45, 50, 52, 56].

Patients’ informational capacity is both a barrier and facilitator to SDM [45, 47, 50–52, 54, 56]. Patients with low or no informational capacity are less likely to be included in SDM by their clinicians. On the other hand, clinicians report being more likely to include patients perceived as having enough informational capacity in decision-making. For some patients, this is due to their past experience in the healthcare system allowing them some sense of what to expect, and therefore increasing self-efficacy [45, 47, 54].

#### Organisation and system

Lack of formal training for SDM is seen as a system-level barrier by clinicians who believe there should be formal training provided to clinicians to ensure all clinicians are working with a similar understanding of what SDM is [46, 48, 50, 51, 56]. It is worth noting that junior clinicians are often trained in SDM, whereas more experienced clinicians may not have received specific training [46, 48].

### Social/professional role and identity

#### Clinicians

“Social/professional role and identity” are important factors for clinicians across different hospital contexts. The way clinicians see their role is an important driver of SDM. Specifically, clinicians who see themselves as educators of patients [46, 49, 50, 52, 53] and/or collaborators with patients [46, 47, 49, 50, 53–55] are more likely to engage their patients in SDM conversations, believing it is their responsibility to help their patients through the decision-making process. The role of interprofessional collaboration is also seen as necessary along the care continuum, with clinicians reporting that consistent messages give patients more time to engage in SDM over multiple conversations with the interprofessional team working together [45, 49–51, 57]. A barrier to SDM is when clinicians see their role as a decision maker for their patients [48, 50, 52, 56, 57] with many reporting being concerned about looking indecisive to their patients [48, 56].

#### Patients

For patients, a facilitator to SDM is having a positive, trusting relationship with their clinician [47, 51, 54, 55].

### Beliefs about capabilities

#### Clinicians

Some clinicians still hold the belief that some patients do not want to be included in decisions about their care [45, 50, 54, 56, 57] and therefore do not include them in the SDM process; those who believe that patients should be involved in decisions about their care actively work to engage them in SDM [47, 50, 52, 55].

#### Patients

Many patients believe they do not have the necessary skills or capabilities to be included in decisions about their care, believing that their clinician knows best and patients should not disagree with them [45, 47, 56, 57]. Others feel it is their responsibility to play an active role in decision-making with their clinician, due to either past experience in the healthcare system or confidence in their own lived experience [45, 47, 53, 54].

### Environment, context, and resources

#### Clinicians

There are a range of “Environmental context and resource” factors that inhibit clinicians practising SDM. Barriers identified include lack of time, busy and noisy ward surroundings, lack of private spaces, and the presence of family members. Lack of time is cited in nine of the fourteen studies [44, 45, 47–50, 56, 57] with clinicians and patients citing ongoing interruptions, overall workload (including administrative tasks), and acuity of other patients. Additionally, busy and noisy ward environments also make it difficult for clinicians and patients to engage in SDM [47, 48, 50, 51, 53, 56], with some specifically citing lack of private spaces to conduct SDM conversations [47, 50, 51, 56]—for example patients in emergency departments placed in hallways due to lack of space [47, 48, 56]. The presence of family members is seen as a barrier by some clinicians [49, 51, 56] who feel their presence can create additional complexities in decision-making; conversely, other clinicians recognise that having family members present provides an additional resource for patients to discuss options [45, 47, 50, 54] or even to translate [54].

#### Patients

Patient characteristics include those that are difficult to change or modify and can either work as barriers or facilitators to SDM. These include low socio-economic status, multiple comorbidities, language barriers, and past negative healthcare experiences. Conversely, patients who have higher socio-economic status, higher education level, and past positive experiences with healthcare are more likely to engage in and be engaged in SDM [45–51, 55, 56].

The presence of a carer or family member may provide support for some patients during their time in the hospital included during the SDM process. Patients report feeling that they can rely on their carer/family member to help provide clinicians with their preferences and as a sounding board during decision-making conversations [45, 47, 49, 51, 53].

#### Organisation and system

System-level factors that inhibit SDM included lack of or fragmented availability of clinical guidelines that support the use of SDM [44, 46, 55, 57]. Some see the solution to this as changing clinical guidelines to support the use of SDM by explicitly mentioning SDM [44, 46, 55, 57] and making locally based, context-specific SDM implementation evidence [44, 46]. Noisy and busy ward environment [47, 48, 50, 51, 53, 56] and a lack of private space to conduct SDM conversations [47, 48, 50, 51, 56] are also reported as potential organisational-related factors that could change the likelihood of conducing SDM conversations.

**Table 3:** Summary of review findings for dominant themes

Summary of review findings Studies contributing to the review finding ***Illustrative quote***	CERQual assessment of confidence in the evidence
**Knowledge:**	
**Clinician-related factors: Not knowing what SDM is,** a number of clinicians have either no knowledge of or an incorrect working definition of what SDM is [52] [55] [46] [57] *“[Some clinicians] understood SDM as the professional collaboration between care providers prior to discussing the options with the patient. We make the decision as a team whether or not the patient should go for a cath. I don’t frequently give patients—if I’m sending a patient, if I make the decision that this is appropriate, then we go through the risks and benefits.”* [52]	High confidence
**Patient-related factors: Patients who are well informed** prior to the SDM conversation, report feeling able to engage in SDM conversations with their clinician, (especially those who are able to understand the risks and benefits of their options) [45, 47, 50–54, 57] [45, 47, 51–53] *“Additional patient behaviours that take place outside the consultation, including gathering medical information and preparing for the consultation were also identified as important.”* [54]	High Confidence
**Patient-related factors: Lack of knowledge of risk** of different treatment options is seen as a barrier for both clinicians in trying to explain options and patients trying to understand their different treatment options [44, 45, 50, 52, 53, 56] *“I had no knowledge and I still don’t have much knowledge about what the complications could have been.”* [52] *I think after the procedure the nurse or some knowledgeable person should have walked me through what was done, how, when, why, and where. I really wasn’t informed”* [52]	High confidence
**Skills:**	
**Clinician-related factors: Communication skills** were identified by clinicians as necessary to elicit patient preferences and enable SDM conversations [45–48, 50–53, 56, 57] *“Yeah, I mean. I think any sort of training in communication and helping with choices and that sort of thing is probably helpful.”* [51]	High confidence
**Clinician-related factors: Formal training** was identified as a facilitator (lack of a barrier), with clinicians noting training would provide them with confidence to know they were doing SDM correctly. [44, 48–51, 55, 56] *“I’ve not had any formal training in it… I’m very comfortable in it but I don’t know if it matches with the techniques that others use.’ ‘I’ve done it more than most and therefore am comfortable, not that I’m doing it right or anything”* [56]	Moderate confidence
**Clinician-related factors: Trust in one’s own clinical expertise and past experience** were facilitators for clinicians, who felt past experience helped them increase their clinical skills, confidence and awareness of their own limitations [47, 48, 55–57] *“I think I do that [SDM] a lot more now than I did when I started. When I started it was kind of like . . . you follow protocols and evidence-based medicine and all these things, and [back] then I just didn’t feel comfortable swaying from some of those things, and now I feel like my instincts are a piece of that puzzle, of using the evidence-based medicine and things like that. If that’s getting me to a point where it’s 50/50 or 60/40 in that range, then I just start talking to [the] patient and figure out ‘What are you trying to get?”’* [48]	Moderate confidence
**Patient-related factors: Patients’ informational capacity** is both a barrier and facilitator of SDM with those lacking informational capacity less likely to be engaged in SDM and those with informational capacity being included in informed decisions (for some this was due to past experience within the healthcare system) [45, 47, 50–52, 54, 56] ( [45, 47, 54]) *“Their ability to understand that they really, really need to come back if something different happens, is really important for me to involve them in the decision making process.”* [56]	High confidence
**Organisation- and system-related factors: Lack of formal training for SDM** is seen as a system-level barrier by clinicians who believe there should be formal training provided to clinicians [46, 48, 50, 51, 56] *“Participants stressed that training will be vital in order to overcome the belief among clinicians that applying SDM does not differ much from their current practice.”* [46]	Moderate confidence
**Environmental Context and Resources:**	
**Clinician-related factors: Lack of time** was a major barrier due to numerous interruptions, overall workload (including administrative tasks), and competing priorities including acuity of other patients [44, 45, 47–50, 53, 56, 57] *“I think everyone recognizes that we as physicians and extenders and team members don’t have the time to really spend to help patients make decisions that are good for them”* [57]	High confidence
**Clinician-related factors: Busy and noisy ward** environments also make it difficult for clinicians and patients to engage in SDM [47, 48, 51, 53, 56] *“What I’m seeing recently is that the patients want to be listened to, but the environment on the ward is so difficult and so chaotic. We are there trying to listen to their expectations, the way they feel so we can try to change something but if the environment remains the same is very difficult to do’”* [52]	Moderate confidence
**Clinician-related factors: Lack of private spaces** to conduct SDM conversations is a barrier for clinicians and patients, especially those placed in hallways when there are not enough available beds in emergency departments [47, 48, 50, 51, 56]*“Having a real conversation in the hallway, it’s not private…can’t sit down…”* [56]	Moderate confidence
**Clinician-related factors: Presence of family members** is seen as both a barrier and facilitator for clinicians. Some clinicians report additional complexity, while others see family members as a resource for patients that enables SDM conversations. [49, 51, 56] (barrier) [45, 47, 49, 50, 52, 53] (facilitator) *“Obviously the more people are involved, the more points of conflict there are and the more there is to be negotiated you know but by not involving them, you don’t take that complexity away necessarily”* [51] *“Sometimes, doctors will give you information and just like the tip of the iceberg. I like to have my daughter along when we’re talking to a doctor because she has some very pointed questions that she puts to them. I get a lot of information through my daughter’s questioning.”* [52]	Moderate confidence
**Patient-related factors: Patient characteristics barriers reported include** low socio-economic status, multiple comorbidities, English (or countries most popular language) as a second language, and past negative healthcare experiences. Whereas patients who had higher socio-economic status, higher education level, and past positive experiences with healthcare report being more likely to engage in and be engaged in SDM [45–51, 55, 56] *“If there’re huge language barriers, unfortunately even with a translator, sometimes those nuances are lost.’ ‘They think it’s because of who they are, that they don’t have insurance, that’s why we’re not admitting them… I think (with) that specific population I have a very hard time doing (shared) decision making with”* [56]	Moderate confidence
**Patient-related factors: Presence of carer or family members** provide support for patients going during decision-making and treatment, patients report feeling they can rely on their carer/family to encourage SDM with their clinician [45, 47, 49–51, 53] *“One patient asked her daughter accompanying her during an inpatient consultation, ‘Isn‘t that good [treatment]?’ in order to reassure herself.”* [53] **“***It’s hard to be an advocate for yourself when you’re by yourself. It would be easier if you had someone here for you*.” [47]	Moderate confidence
**Organisation- and system-related factors: Changing clinical guidelines to promoted SDM** is reported by clinicians and other stakeholders as being one way in which the system could be changed to facilitated SDM [44, 46, 55, 57] *“I really like hospital guidelines, especially if they’re done well where they don’t limit me, yet they give me kind of a something to stand on ... give me protection for what I think is right even though there is a small amount of risk involved in doing it.”* [55]	High confidence
**Organisation- and system-related factors: Noisy or busy ward environment** also makes it difficult for clinicians and patients to engage in SDM [47, 48, 50, 51, 53, 56] *“A number of clinicians mentioned that due to the ward being so busy, they were sometimes unable to find a space to sit down with the patient and have a conversation”* [51]	Moderate confidence
**Organisation- and system-related factors: Lack of private spaces** to conduct SDM conversations is a barrier for clinicians and patients, especially those placed in hallways when there are not enough available beds in emergency departments [47, 48, 50, 51, 56] *“This is not a hallway thing”* [56]	Moderate confidence
**Social/Professional Role and Identity**	
**Clinician-related factors: Clinician’s perceived role as educator.** Clinicians who saw their role as educators (and/or collaborators) reported being more likely to engage in SDM with their patients, proving information to patients before helping them through the decision-making process [46, 47, 49, 50, 53–55] *“making sure that they have information on the available treatment alternatives…within drug treatment, there are a number of different options available. Giving them those options and that independence of making a choice, that’s helpful as well.”* [50]	High confidence
**Clinician-related factors: Clinicians perceived role as decision maker** is a barrier for clinicians who feel it is their responsibility to make decisions for their patients, with a number citing concerns over looking indecisive to their patients. [48, 50, 52, 56, 57] *“I think that people want to know that the doctor that they talked to had found something or was confident in this is what’s going on, and so I think that if I don’t do a good job, of that or come in too shared decision-making-oriented, where ‘maybe it’s this, maybe it’s that,’ …I don’t want to sound too wishy-washy”* [56]	High confidence
**Clinician-related factors: Interprofessional collaboration** is seen as a key facilitator of SDM, clinicians feel it is crucial that all members of the care team are communicating the same message to the patient to enable ongoing SDM across multiple conversations with members of the team [45, 49–51, 57] *“... you’ve got multiple doctors or multiple specialists involved who have vying opinions in relation to what’s occurring ... what can happen is it can lead to medications being changed quite rapidly .. . which in a patient’s mind creates this lack of confidence ... ”* [50]	Moderate confidence
**Patient-related factors: Patients having a trusting relationship** with their clinician was seen to facilitate SDM [47, 50, 51, 54, 55] *“patients emphasized that being patient, having some trust in advance and giving doctors and therapies a try might be helpful”* [54]	High confidence
**Beliefs about capabilities:**	
**Clinician-related factors: Belief that the patient does not want to be involved in decision-making** is a barrier for clinicians who assume their patient does not want to engage is SDM. [45, 50, 54, 56, 57] (barrier) *“suggesting that clinicians presume that many patients will not benefit from SDM or do not wish to take part.”* [45] *“‘Sometimes patients just want to be told what to do. “Others have clearly expressed to me that they don’t want to have any part in that decision, ‘(You’re) the goddamn doctor, why don’t you make a decision?’”* [56] Conversely a number of clinicians hold the **belief that patients should be involved** in decisions about their care and actively work to engage them [47, 50, 52, 55](facilitator) ***“****I think it’s super important some of the questions that you pose for patients to think about, like is this congruent with quality of life. I’ve been there at the eleventh hour and people have to make decisions about what they want and don’t want, and my hope is for them to have a decision aid going into this.”* [57]	High confidence
**Patient-related factors: Patients belief that they should not disagree** with their clinician is a barrier to SDM [45, 47, 56, 57] *“How can you make a decision when you’re not an expert?...The bottom line is I am not a doctor.”* [47]	High confidence
**Patient-related factors: Patients beliefs that they should be included** in decisions about their own care, either due to past experience in the healthcare system and/or confidence in their own knowledge of their lived experience [45, 47, 53, 54] *“This is my life, and I need to be able to make that decision because they are not the one who is suffering. I am the one that is suffering.”* [47]	Moderate confidence

### Additional factors not shared across clinician-, patient-, organisation-, system-, and stakeholder-related factors

#### Additional clinician-related factors

“Beliefs about consequences” are other factors with high confidence found in the review findings (Table 3). Clinicians report not engaging in SDM when they believe there may be a negative outcome for their patient either due to the acuity of the patient’s disease and treatment options [45, 52, 55, 56], for example in cardiology or the emergency department, or the potential risks of making “the wrong decision” [47, 52, 56, 57].

**Table 4:** Summary of review findings for clinician-related factors

Summary of review findings for additional clinician-related factors ***Illustrative quote***	CERQual assessment of confidence in the evidence
**Intentions:**	
**Predetermined treatment decision** A number of clinicians decide on the treatment plan before engaging in decision-making conversations, with the intention of “selling” the patient on the treatment they have selected for them [47, 48, 50–52, 55, 56] *“In most cases, the physicians made the treatment decisions. Either one physician made the treatment decision by himself or several physicians made medical decisions jointly (especially in inpatient wards). For example, one observer noted, …‘Most decisions during ward rounds [at inpatient wards] are taken in front of the computer before entering the patient’s room’.”* [53]	High confidence
**Beliefs about Consequences:**	
**Negative Outcomes** Clinicians reported not engaging in SDM when they are concerned about the potential of a negative outcome, sometimes this is due to the acuity of the decision or the potential risks [45, 52, 55, 56] [47, 52, 56, 57] *“[Interviewer: Tell about times you don’t use SDM?] ‘STEMIs [ST segment elevation myocardial infarction], I’m not asking a lot of questions, I’m going forward.’”* [56]	High confidence

#### Additional patient-related factors

For patient-related factors other dominant themes include, “Emotion”, and “Social/Professional Role and Identity” (Table 5). Fear of negative outcomes is a barrier for patients, with some reporting they purposely do not engage in decisions about their care for fear that doing so would result in a negative outcome by making the “wrong” decisions [45, 49, 55, 56]; however, this review finding has low confidence.

**Table 5:** Summary of review findings for patient-related factors

Summary of review finding for additional patient-related factors ***Illustrative quote***	CERQual assessment of confidence in the evidence
**Emotion:**	
**Fear of negative outcomes** is a barrier to SDM for patients, with some patients reporting not wanting to engage because of a fear that doing so will result in negative outcomes and believing there is a right or wrong decision to be made [45, 47, 49, 55, 56] *“Maybe sometimes I’m afraid to say something because it will be something worse than I think it is. [You don’t want to bring it up because you’re afraid you might get bad news?] Yes.”* [47]	Low confidence

#### Additional organisation- and system-related factors

“Social influence” is one of the most reported facilitators of SDM at the organisational level. The culture of the organisation is seen as crucial to the success of SDM being used by clinicians (Table 6). Clinicians and other stakeholders report when there is a clear organisational shift toward SDM, it is easier to facilitate SDM in practice [46, 48, 55–57]. Additionally, when leaders are seen as engaging in SDM, participants report feeling supported to try SDM, and for health service decision makers and administrators, leadership support was key in promoting the implementation of their SDM programme with clinicians [44, 46, 48, 57].

**Table 6:** Summary of review findings for organisation- and system-related factors

Summary of review finding for additional organisation- and system-related factors ***Illustrative quote***	CERQual assessment of confidence in the evidence
**Social Influence:**	
**Culture of the organisation** is seen as an important organisational-related factor with participants reporting feeling more supported to engage in SDM when it is clear that their organisation supports them to do so. [46, 48, 55–57] *“‘What is more powerful is the culture of the institution, right? Where I trained before, at a county hospital, we didn’t admit anybody for chest pain… you’d talk to them about the risk and … that was what the institution, and…the population, expected”* [56]	Moderate confidence
**Leaders engaging in SDM** is seen as an important organisational-related factor with participants reporting feeling more supported to engage in SDM when it is clear that their leaders use and support the use of SDM [44, 46, 48, 57] *“I know [surgical director] feels pretty strongly that it’s a good tool and was the one who pushed the initial use of it . . . he says to us “make sure you’re using this.”* [57]	High confidence

#### Stakeholder-related factors beyond the patient-clinician dyad

Only three of the 14 included studies included stakeholders outside the patient-clinician dyad [45, 46, 57]; as such, these findings should be interpreted with caution. Stakeholder-related dominant themes were “Knowledge”, “Social/professional role and influence”, “Environment, context and resources”, and “Social influence”. Stakeholders see their role as one of facilitator—monitoring SDM implementation [46] and encouraging implementation of SDM through education of clinicians and patients [57], while anticipating personnel and budget requirements to ensure ongoing implementation efforts [46]. The importance of having site champions and other leaders who are willing to encourage the workforce to engage in SDM delivery was recognised [46, 48, 57].

## Discussion

This is the first known systematic review of barriers and facilitators to implementing SDM in hospital settings that aimed to examine barriers and facilitators including and beyond the patient-clinician dyad. Using the Best Fit Framework Synthesis, this review builds on previous work by extracting data to previous reviews taxonomies [14, 15], then extracted to the TDF. The most salient TDF domains were “Knowledge” and “Skills”, “Environmental Context and Resources”, “Social/Professional Role and Identity”, and “Beliefs about Capabilities”.

Six electronic databases were searched, which allowed for the most relevant articles to be picked up by the search strategy. Additionally, a comprehensive search was undertaken as reflected by the n = 8724 articles screened for their title and abstract and a further n = 520 reviewed in full text. Only English language articles were included in the review and grey literature search was not conducted which are limitations. Implementation programmes may have been missed that have not been published in academic literature. However, an additional search of systematic reviews included in the last stage of screening was undertaken to ensure no relevant peer-reviewed articles meeting inclusion and exclusion criteria were missed. Two reviewers conducted data assessment for title and abstract screening and full-text inclusion; however, only one reviewer conducted data extraction for the included studies. The review methodology attempted to mitigate bias by having a second reviewer assess over 10% of the studies, and when there was any doubt, the first reviewer asked for the second reviewer’s input until consensus was reached. One identified study was not included as the study author did not respond to requests for information pertaining to eligibility criteria. The current review includes and synthesises studies published since 2008, building on the last substantive review conducted by Légaré et al.’s (2008) [14] review of clinician’s barriers and facilitators to SDM. The exclusion of articles prior to 2008 is a limitation; however, the current review aims to focus on articles produced during the exponential growth in the field since 2008 [25].

At the study level, some studies did not adequately report on the relationship between researcher and participants [44, 48, 50, 52, 53] while two studies were of low quality [44, 48], not attributing findings to participants or using quotes. This was considered when assessing the overall confidence in the evidence. At the review level, there were minor concerns with coherence. Adequacy and relevance tended to be of no or very minor concern, except for findings including studies by Schoenfeld et al. [47, 54, 55] were of moderate or low confidence.

Exploration of barriers and facilitators to SDM from an organisational and system level is still in its infancy [13, 18, 19]. This review adds to Scholl et al.’s [13] scoping review of organisational- and system-level characteristics that influence the implementation of SDM by going beyond the patient-clinician dyad. Scholl et al.’s [13] scoping review of influential characteristics at the organisation and system levels found that factors associated with the success of SDM implementation include adequate resourcing, setting of SDM as a priority, integration of SDM into teams and workflow, and cultural and organisational leadership, whereas at the system level, factors include clinical guidelines, incentives, education, licencing, culture, and policy. The present study corroborated the same factors reported by Scholl et al. [13] at the organisation and system level; however, one main difference in this study was the call for changing organisational- and system-level guidelines to promote and allow for the use of SDM in practice.

The addition of individual-level factors in this review mapped to the TDF shows that clinician barriers to SDM, such as a lack of knowledge and skills to practice SDM and a belief that SDM is not being used by colleagues, may be changed through the use of changing organisation- and system-level guidelines. It is important to bear in mind that not all factors have been reported by the population themselves—for example clinicians, rather than patients, reported that patients have a poor understanding of risk. This should be taken into account when interpreting findings.

Further differences exist between this review’s findings for clinician-related factors and organisational- and system-related factors when compared to existing literature. For example, Légaré et al. (2008) [14] reported barriers such as time constraints, patient characteristics, and the clinical situation. This review found the most frequently reported barriers were “Skills”, including a lack of training in communication and SDM and trust in one’s own clinical ability. Additionally, the busy and noisy environments (for example, wards) and a lack of private spaces to conduct SDM is a barrier not previously reported and which is less likely to be encountered in non-hospital settings. This demonstrates how focusing on hospital settings has built upon the understanding of SDM.

Many similarities were identified between existing literature on primary and secondary care settings and that of tertiary care explored in this review. The lack of time is consistent across settings with clinicians reporting struggling to fit SDM conversations into busy work schedules [13–16]. Additionally, many of the “Beliefs About Capabilities” were shared across settings, for example clinicians’ belief that patients do not want to be included in decisions about their care or patient beliefs that they should not disagree with their clinicians’ recommendations [14, 15]. Barriers related to “Knowledge” and “Skills” were also seen across settings with clinicians’ lack of awareness of the correct definition of SDM [14]. Support of SDM also varied in line with existing literature with some clinicians in favour of and some not in favour of using SDM, depending on the perceived feasibility of including patients given the clinical context and patient characteristics [58]. For patient-related factors, this review did not find any differences in results to that of Joseph-Williams et al. [15], except for the stressful environment due to the busy and noisy ward environment with little private spaces in which to conduct a SDM conversation.

This review reinforces previous research stating that SDM research in tertiary settings and beyond the patient-clinician dyad is in its infancy [13]. There were few (N = 14) articles that looked at the hospital inpatient setting, and only three of these included stakeholders in addition to the patient-clinician dyad. The perspectives of these additional stakeholders illuminate factors not reported by patients and clinicians such as facilitating implementation strategies, budgets and personnel requirements. These insights may further support the implementation of SDM by enabling consideration of factors beyond the patient and clinician, but which are critical to ensuring that patients and clinicians have an opportunity to participate in SDM.

Results from this study show that the majority of barriers and facilitators to implementing SDM in practice are shared across primary, secondary, and tertiary care. However, there are some contextual factors that make SDM even more difficult in tertiary care, including busy and noisy ward environments and a lack of private spaces in which to conduct SDM conversations.

Given the small yield in this review, additional studies in tertiary settings and beyond the clinician-patient dyad are needed. These may further facilitate the exploration of organisation-and system-level characteristics that can be the target of future implementation of SDM.

This review focused on SDM in developed countries. Low and middle-income countries may have additional barriers and facilitators specific to their context. Further research is needed to explore SDM implementation in low and middle-income countries.

This review carries a number of implications for patients, clinicians, and other stakeholders. Patients who are able to prepare for SDM encounters may experience fewer barriers. For example, patients who believe they should be included in decisions, are well informed prior to the SDM encounter, and have adequate informational capacity report feeling better able to engage in SDM conversations while clinicians are more likely to engage them. Additionally, patients who have a carer or family member present, and a trusting relationship with their clinician report feeling supported through the SDM process. It is important to note that these factors are difficult for patients to alter themselves, especially in the high-stress context of the tertiary healthcare setting where they may be acutely ill and under time pressures. Therefore, an important implication is the need for clinicians and other stakeholders to facilitate SDM. Clinicians should consider their underlying beliefs about patients prior to excluding them from the SDM process; and can also facilitate SDM through SDM and communication skills training, interprofessional collaboration, and promoting SDM among colleagues and junior clinicians. Healthcare decision makers and administrators can facilitate SDM by providing an enabling environment—quiet, private spaces for SDM conversations; time for SDM conversations in clinicians’ busy workloads; and ongoing training for clinicians in SDM and communication. Government policy makers can facilitate SDM through updating clinical guidelines to include recommendations to embed SDM into routine practice and provide training for all clinicians (both junior and senior) in SDM and communication.

## Conclusion

This systematic review explored barriers and facilitators to SDM in the hospital setting and from the perspective of those within and beyond the clinician-patient dyad. A range of barriers and facilitators across individual, organisational, and system levels were reported. Based on analysis using the TDF, the dominant themes were “Knowledge”, “Skills”, “Environmental Context and Resources”, “Social/Professional Role and Identity”, and “Beliefs about Capabilities”. Barriers specific to hospital setting were noisy and busy ward environments and lack of private spaces in which to conduct SDM conversations. Based on this review findings, healthcare organisations and governments should consider the role of additional stakeholders outside the patient-clinician dyad. Additionally, those working to implement SDM in the hospital setting should consider the contextual factors that are different from those seen in primary and secondary care. Further research is needed to explore SDM implementation in hospital settings, while including the perspectives of additional stakeholders to explore how barriers may be overcome and facilitators enhanced.

## Supplementary Information

**Additional File 1.** Search String Example (OVID Medline search example).

**Additional File 2.** Study Characteristics of Included Studies (table containing author, year, country, study design, participants and implementation intervention for each of the included studies).

**Additional File 3.** Modified Critical Appraisal Skills Programme (CASP) Tool (table containing results of the CASP tool for each of the included studies).

**Additional File 4. **Barriers and Facilitators by frequency of citation to SDM Mapped to the TDF for Multiple Stakeholders (table containing frequency of citations of SDM mapped to the TDF where *n*=number of citations).

**Additional File 5.** PRISMA Checklist for Reporting Systematic Reviews (checklist items and corresponding page references for PRISMA).

## Data Availability

The datasets used and/or analysed during the current study are available from the corresponding author on reasonable request.

## Abbreviations

SDM: Shared decision-making
PCC: Patient-centred care
TDF: Theoretical Domains Framework
BFFS: Best Fit Framework Synthesis
CASP: Critical appraisal skills programme
HSA: Health service administrator
HSDM: Health service decision maker
GPM: Government policy maker

## References

[1] Charles C, Gafni A, Whelan T (1999). Decision-making in the physician-patient encounter: revisiting the shared treatment decision-making model. Soc Sci Med.

[2] Stiggelbout AM, Van Der Weijden T, De Wit MPT, Frosch D, Légaré F, Montori VM (2012). Shared decision making: really putting patients at the centre of healthcare. BMJ.

[3] Müller E, Hahlweg P, Scholl I (2016). What do stakeholders need to implement shared decision making in routine cancer care? A qualitative needs assessment. Acta Oncol.

[4] Elwyn G, Frosch D, Thomson R, Joseph-Williams N, Lloyd A, Kinnersley P (2012). Shared decision making: a model for clinical practice. J Gen Intern Med.

[5] Elwyn GJ, Edwards A, Kinnersley P, Grol R. Shared decision making and the concept of equipoise: the competences of involving patients in healthcare choices. Br J Gen Pract. 2000;50(460):–892 Available from: /pmc/articles/PMC1313854/?report=abstract. PMC1313854 11141876

[6] Charles C, Gafni A, Whelan T (1997). Shared decision-making in the medical encounter: what does it mean? (or it takes, at least two to tango). Soc Sci Med.

[7] Laine C, Davidoff F (1996). Patient-centered medicine. A professional evolution. J Am Med Assoc.

[8] Bot AGJ, Bossen JKJ, Herndon JH, Ruchelsman DE, Ring D, Vranceanu AM (2014). Informed shared decision-making and patient satisfaction. Psychosomatics..

[9] Hughes TM, Merath K, Chen Q, Sun S, Palmer E, Idrees JJ, Okunrintemi V, Squires M, Beal EW, Pawlik TM (2018). Association of shared decision-making on patient-reported health outcomes and healthcare utilization. Am J Surg.

[10] Durand M-A, Carpenter L, Dolan H, Bravo P, Mann M, Bunn F, et al. Do Interventions designed to support shared decision-making reduce health inequalities? A systematic review and meta-analysis. PLoS ONE. 2014;9(4) Available from: www.plosone.org. 10.1371/journal.pone.0094670 PMC3988077 24736389

[11] Dimopoulos-Bick T, Osten R, Shipway C, Trevena L, Hoffmann T (2019). Shared decision making implementation: a case study analysis to increase uptake in New South Wales. Aust Health Rev.

[12] Härter M, Moumjid N, Cornuz J, Elwyn G, van der Weijden T. Shared decision making in 2017: International accomplishments in policy, research and implementation. Z Evid Fortbild Qual Gesundhwes. 2017;123–124:1–5. Available from: 10.1016/j.zefq.2017.05.024 10.1016/j.zefq.2017.05.024 28546053

[13] Scholl I, LaRussa A, Hahlweg P, Kobrin S, Elwyn G (2018). Organizational- and system-level characteristics that influence implementation of shared decision-making and strategies to address them - a scoping review.

[14] Légaré F, Ratté S, Gravel K, Graham ID (2008). Barriers and facilitators to implementing shared decision-making in clinical practice: update of a systematic review of health professionals’ perceptions. Patient Educ Couns.

[15] Joseph-Williams N, Elwyn G, Edwards A. Knowledge is not power for patients: a systematic review and thematic synthesis of patient-reported barriers and facilitators to shared decision making. Patient Educ Couns 2014;94(3):291–309. Available from: 10.1016/j.pec.2013.10.031 10.1016/j.pec.2013.10.031 24305642

[16] Boland L, Graham ID, Légaré F, Lewis K, Jull J, Shephard A, et al. Barriers and facilitators of pediatric shared decision-making: a systematic review. Implement Sci. 2019;14(1). 10.1186/s13012-018-0851-5 PMC6339273 30658670

[17] Chaudoir SR, Dugan AG, Hi Barr C. Measuring factors affecting implementation of health innovations: a systematic review of structural, organizational, provider, patient, and innovation level measures [Internet]. 2013. Available from: http://www.implementationscience.com/content/8/1/22 10.1186/1748-5908-8-22 PMC3598720 23414420

[18] Elwyn G, Frosch DL, Kobrin S (2016). Implementing shared decision-making: consider all the consequences. Implement Sci.

[19] Légaré F, Stacey D, Pouliot S, Gauvin FP, Desroches S, Kryworuchko J, Dunn S, Elwyn G, Frosch D, Gagnon MP, Harrison MB, Pluye P, Graham ID (2011). Interprofessionalism and shared decision-making in primary care: a stepwise approach towards a new model. J Interprof Care.

[20] Michie S, van Stralen M, West R (2011). The behaviour change wheel: a new method for characterising and designing behaviour change interventions. Implement Sci.

[21] French SD, Green SE, O’Connor DA, McKenzie JE, Francis JJ, Michie S (2012). Developing theory-informed behaviour change interventions to implement evidence into practice: a systematic approach using the Theoretical Domains Framework. Implement Sci.

[22] Ofstad EH, Frich JC, Schei E, Frankel RM, Benth JŠ, Gulbrandsen P (2018). Clinical decisions presented to patients in hospital encounters: a cross-sectional study using a novel taxonomy. BMJ Open.

[23] (AIHW). 2.1 How does Australia’s health system work? In: Australia’s Health 2016. Cat. no. A. Canberra; 2016.

[24] Merriam-Webster. Tertiary care [Internet]. Merriam-Webster.com dictionary. [cited 2020 Dec 16]. Available from: https://www.merriam-webster.com/dictionary/tertiary care

[25] Lu C, Li X, Yang K (2019). Trends in shared decision-making studies from 2009 to 2018: a bibliometric analysis. Front Public Health.

[26] Atkins L, Francis J, Islam R, O’Connor D, Patey A, Ivers N (2017). A guide to using the Theoretical Domains Framework of behaviour change to investigate implementation problems. Implement Sci.

[27] Noyes J, Booth A, Cargo M, Flemming K, Garside R, Hannes K, Harden A, Harris J, Lewin S, Pantoja T, Thomas J (2018). Cochrane qualitative and implementation methods group guidance series—paper 1: introduction. J Clin Epidemiol.

[28] Liberati A, Altman DG, Tetzlaff J, Mulrow C, Gøtzsche PC, Ioannidis JPA (2009). The PRISMA statement for reporting systematic reviews and meta-analyses of studies that evaluate health care interventions: explanation and elaboration. PLoS Med.

[29] Cooke A, Smith D, Booth A (2012). Beyond PICO: the SPIDER tool for qualitative evidence synthesis. Qual Health Res.

[30] Veritas Health Innovation. Covidence systematic review software [Internet]. Melbourne, Australia; 2019. Available from: www.covidence.org

[31] Critical Appraisal Skills Programme. CASP qualitative checklist [Internet]. 2018. Available from: www.casp-uk.net

[32] Noyes J, Booth A, Moore G, Flemming K, Tunçalp Ö, Shakibazadeh E. Synthesising quantitative and qualitative evidence to inform guidelines on complex interventions: clarifying the purposes, designs and outlining some methods. BMJ Glob Health.2019;4:893. Available from: 10.1136/bmjgh-2018-000893 10.1136/bmjgh-2018-000893 PMC6350750 30775016

[33] Long HA, French DP, Brooks JM (2020). Optimising the value of the critical appraisal skills programme (CASP) tool for quality appraisal in qualitative evidence synthesis. Res Methods Med Heal Sci.

[34] Lewin S, Booth A, Glenton C, Munthe-Kaas H, Rashidian A, Wainwright M (2018). Applying GRADE-CERQual to qualitative evidence synthesis findings: introduction to the series. Implement Sci.

[35] Lewin S, Bohren M, Rashidian A, Munthe-Kaas H, Glenton C, Colvin CJ, Garside R, Noyes J, Booth A, Tunçalp Ö, Wainwright M, Flottorp S, Tucker JD, Carlsen B (2018). Applying GRADE-CERQual to qualitative evidence synthesis findings-paper 2: how to make an overall CERQual assessment of confidence and create a Summary of Qualitative Findings table. Implement Sci.

[36] Munthe-Kaas H, Bohren MA, Glenton C, Lewin S, Noyes J, Tunçalp Ö, Booth A, Garside R, Colvin CJ, Wainwright M, Rashidian A, Flottorp S, Carlsen B (2018). Applying GRADE-CERQual to qualitative evidence synthesis findings-paper 3: how to assess methodological limitations. Implement Sci.

[37] Colvin CJ, Garside R, Wainwright M, Munthe-Kaas H, Glenton C, Bohren MA, Carlsen B, Tunçalp Ö, Noyes J, Booth A, Rashidian A, Flottorp S, Lewin S (2018). Applying GRADE-CERQual to qualitative evidence synthesis findings-paper 4: how to assess coherence. Implement Sci.

[38] Glenton C, Carlsen B, Lewin S, Munthe-Kaas H, Colvin CJ, Tunçalp Ö, Bohren MA, Noyes J, Booth A, Garside R, Rashidian A, Flottorp S, Wainwright M (2018). Applying GRADE-CERQual to qualitative evidence synthesis findings-paper 5: how to assess adequacy of data. Implement Sci.

[39] Noyes J, Booth A, Lewin S, Carlsen B, Glenton C, Colvin CJ, Garside R, Bohren MA, Rashidian A, Wainwright M, Tunςalp Ö, Chandler J, Flottorp S, Pantoja T, Tucker JD, Munthe-Kaas H (2018). Applying GRADE-CERQual to qualitative evidence synthesis findings-paper 6: How to assess relevance of the data. Implement Sci.

[40] Carroll C, Booth A, Cooper K (2011). A worked example of “best fit” framework synthesis: a systematic review of views concerning the taking of some potential chemopreventive agents. BMC Med Res Methodol.

[41] Carroll C, Booth A, Leaviss J, Rick J (2013). “best fit” framework synthesis: refining the method. BMC Med Res Methodol.

[42] Booth A, Carroll C (2015). How to build up the actionable knowledge base: the role of “best fit” framework synthesis for studies of improvement in healthcare. BMJ Qual Saf.

[43] Cane J, O’Connor D, Michie S (2012). Validation of the theoretical domains framework for use in behaviour change and implementation research. Implement Sci.

[44] Allen KM, Dittmann KR, Hutter JA, Chuang C, Donald ML, Enns AL (2020). Implementing a shared decision-making and cognitive strategy-based intervention: knowledge user perspectives and recommendations. J Eval Clin Pract.

[45] Barrett TW, Rising KL, Bellolio MF, Hall MK, Brody A, Dodd KW (2016). The 2016 Academic Emergency Medicine Consensus Conference, “Shared Decision Making in the Emergency Department: Development of a Policy-relevant Patient-centered Research Agenda” diagnostic testing breakout session report. Acad Emerg Med.

[46] van Veenendaal H, van der Weijden T, Ubbink DT, Stiggelbout AM, van Mierlo LA, Hilders CGJM (2018). Accelerating implementation of shared decision-making in the Netherlands: An exploratory investigation. Patient Educ Couns.

[47] Schoenfeld EM, Goff SL, Downs G, Wenger RJ, Lindenauer PK, Mazor KM (2018). A qualitative analysis of patients perceptions of shared decision making in the emergency department: “Let Me Know I Have a Choice”. Acad Emerg Med.

[48] Schoenfeld EM, Goff SL, Elia TR, Khordipour ER, Poronsky KE, Nault KA, Lindenauer PK, Mazor KM (2018). A qualitative analysis of attending physicians’ use of shared decision-making: implications for resident education. J Grad Med Educ.

[49] Pyl N, Menard P (2012). Evaluation of nurses’ perceptions on providing patient decision support with cardiopulmonary resuscitation. ISRN Nurs.

[50] Chong WW, Aslani P, Chen TF (2013). Shared decision-making and interprofessional collaboration in mental healthcare: a qualitative study exploring perceptions of barriers and facilitators. J Interprof Care.

[51] Giacco D, Mavromara L, Gamblen J, Conneely M, Priebe S (2018). Shared decision-making with involuntary hospital patients: a qualitative study of barriers and facilitators. BJPsych Open.

[52] Grant EV, Summapund J, Matlock DD, Vaughan Dickson V, Iqbal S, Patel S (2020). Patient and cardiologist perspectives on shared decision making in the treatment of older adults hospitalized for acute myocardial infarction. Med Decis Mak.

[53] Hahlweg P, Härter M, Nestoriuc Y, Scholl I (2017). How are decisions made in cancer care? A qualitative study using participant observation of current practice. BMJ Open.

[54] Hamann J, Kohl S, McCabe R, Bühner M, Mendel R, Albus M (2016). What can patients do to facilitate shared decision making? A qualitative study of patients with depression or schizophrenia and psychiatrists. Soc Psychiatry Psychiatr Epidemiol.

[55] Schoenfeld EM, Goff SL, Elia TR, Khordipour ER, Poronsky KE, Nault KA (2016). The Physician-as-stakeholder: an exploratory qualitative analysis of physicians’ motivations for using shared decision making in the emergency department. Acad Emerg Med.

[56] Schoenfeld EM, Goff SL, Elia TR, Khordipour ER, Poronsky KE, Nault KA (2019). Physician-identified barriers to and facilitators of shared decision-making in the emergency department: an exploratory analysis. Emerg Med J.

[57] Thompson JS, Matlock DD, Morris MA, McIlvennan CK, Allen LA (2018). Organic dissemination and real-world implementation of patient decision aids for left ventricular assist device. MDM policy Pract.

[58] Pollard S, Bansback N, Bryan S. Physician attitudes toward shared decision making: a systematic review. Patient Educ Couns 2014;98(9):1046–1057. Available from: 10.1016/j.pec.2015.05.004 10.1016/j.pec.2015.05.004 26138158

