# Supplementary material for: Barriers and facilitators to shared decision-making in hospitals from policy to practice: a systematic review
Source: Implement Sci. 2021 Jul 31;16:74. doi: 10.1186/s13012-021-01142-y (PMC8325317; doi:10.1186/s13012-021-01142-y)
Supplement: Supplementary file 1 — Additional File 1. Search String Example (OVID Medline search example). [file 13012_2021_1142_MOESM1_ESM.docx]

**Search String Example**

OVID Medline

1. (shared decision or sharing decision or informed decision or informed choice or decision aid).ti,ab.
2. Decision making, shared/ or clinical decision making/ or exp decision making/ or Decision support technique/
3. (decision making or decision support or choice behavio?r*).ti,ab. or ((decision* or choice*) adj2 (making or support* or behavio?r*)).ti.
4. 2 or 3
5. patient participation/
6. (patient participation or consumer participation or patient involvement or consumer involvement).ti,ab. or ((patient* or consumer*) adj2 (involvement or involving or participation or participating)).ti.
7. 5 or 6
8. 4 and 7
9. 1 or 8
10. (implement* or interven* or model* or pract*).mp. [mp=title, abstract, original title, name of substance word, subject heading word, floating sub-heading word, keyword heading word, organism supplementary concept word, protocol supplementary concept word, rare disease supplementary concept word, unique identifier, synonyms]
11. 9 and 10
12. "Attitude of Health Personnel"/ or Health Knowledge, Attitudes, Practice/ or Patient acceptance of health care/ or attitude/ or motivation/ or drive/ or goals/ or intention/ or exp power, psychological/
13. (attitude* or belief* or opinion* or preference* or motivat* or barrier* or facilitat* or drive* or obstacle*).ti,ab.
14. 12 or 13
15. 11 and 14
16. (interview: or experience:).mp. or qualitative.tw. or (descript* adj1 stud*:).mp. or editorial*:.mp. or (cohort adj1 stud*:).mp. or (observation* adj1 stud*).mp. or evaluat*.mp. or review*.mp. or focus group*.mp. [mp=title, abstract, original title, name of substance word, subject heading word, floating sub-heading word, keyword heading word, organism supplementary concept word, protocol supplementary concept word, rare disease supplementary concept word, unique identifier, synonyms]
17. 15 and 16
18. limit 17 to (english language and yr="2008 -Current")
