# Supplementary material for: Barriers and facilitators to shared decision-making in hospitals from policy to practice: a systematic review
Source: Implement Sci. 2021 Jul 31;16:74. doi: 10.1186/s13012-021-01142-y (PMC8325317; doi:10.1186/s13012-021-01142-y)
Supplement: Supplementary file 2 — Additional File 2. Study Characteristics of Included Studies (table containing author, year, country, study design, participants and implementation intervention for each of the included studies). [file 13012_2021_1142_MOESM2_ESM.docx]

**Study Characteristics of Included Studies**

| **Study ID**  **(reference)** | **Author (year), Country** | **Study Design** | **Context, Setting (Decision)** | **Participants (Position)** | **Implementation Intervention** |
| --- | --- | --- | --- | --- | --- |
| 1  (44) | Allen et al., (2020), Canada | Focus Groups | Stroke Rehabilitation, inpatient rehabilitation (goal setting) | *N*=8, site champions from each of the 5 intervention sites (HCP) | CO-OP knowledge translation: 2-day training workshop with an interprofessional team, followed by a 4-month implementation period. During the implementation period, six site visits were conducted by the implementation facilitators. A consolidation session was conducted following the implementation period. |
| 2  (45) | Barrett et al., (2016), United States | Conference breakout session | Emergency Department, Emergency Department (diagnostic testing) | *N*=35, Attendees of the 2016 Academic Emergency Medicine Consensus Conference breakout session (HSA, GPM, other stakeholders) | SDM in general |
| 3  (50) | Chong et al., (2013), Australia | In-depth semi-structured interviews | Acute Mental Health, inpatient mental health (medication adherence in patients with depression and mental health in general) | *N*=31 Health care providers working across hospital (n=19) and primary care (n=12) settings including n=4 psychiatrists, n=4 GPs, n=11 pharmacists, n=7 mental health nurses, n= 5 OT/Psychologists/SW  "All had a variety of experience across mental health units in hospitals, GP practices, hospital pharmacy departments and community pharmacies."  (HCP) | SDM in general |
| 4  (51) | Giacco et al., (2018), United Kingdom | Focus Groups  In-depth semi-structured interviews | Acute Mental Health, inpatient mental health involuntary admission, (many) | *N*=34 (n=18 patients, diagnosis - psychotic disorder = 9, mood disorder=7, substance misuse disorder = 2)  n=16 clinicians (nurse=6, psychiatrists = 6, clinical psychologists =4))  (HCP, Patients) | SDM in general |
| 5  (52) | Grant et al., (2020), United States | In-depth semi-structured interviews | Cardiology, inpatient, (Myocardial Infarction invasive coronary angiography vs. conservative management with medication alone) | *N*=40 (n=20 patients, n=20 cardiologists)  (HCP, Patients) | SDM in general |
| 6  (53) | Hahlweg et al., (2016), Germany | Passive Observation | Oncology, inpatient and outpatient,  (Many) | *N*=108 patients (n=54 in outpatient, n= 54 inpatients)  (HCP, Patients) | ‘three talk collaborative deliberation model’ of  SDM by Elwyn |
| 7  (54) | Hamann et al., (2016), Germany | Focus Groups | Acute Mental Health, inpatient and outpatient  (Many) | *N*= 33, n=16 patients across 4 focus groups, n=17 physicians across 3 focus groups  (HCP, Patients) | SDM in general |
| 8  (49) | Pyl & Menard, (2012), Canada | Before/After Survey  Observation | Acute Monitor Area Unit, inpatient, (CPR status) | *N*=21 nurses, (only n=16 completed the both pre and post-intervention questionnaire)  (HCP) | CPR Status Patient Decision Aid and brief educational session |
| 9  (55) | Schoenfeld et al., (2016), United States | In-depth semi-structured interviews | Emergency Department, Emergency Department, (Many) | *N*=15 Emergency Physicians  (HCP) | SDM in general |
| 10  (48) | Schoenfeld et al., (2018), United States | In-depth semi-structured interviews | Emergency Department, Emergency Department, (Many) | *N*=15 Emergency Physicians  (HCP) | SDM in general |
| 11  (47) | Schoenfeld, Goff, Downs, et al., (2018), United States | In-depth semi-structured interviews | Emergency Department, Emergency Department, (Many) | *N*=26, n= Patients who were deemed clinically stable (or their proxy)  (Patients) | SDM in general |
| 12  (56) | Schoenfeld et al., (2019), United States | In-depth semi-structured interviews | Emergency Department, Emergency Department, (Many) | *N*=15 Emergency Physicians  (HCP) | SDM in general |
| 13  (57) | Thompson et al., (2018), United States  Canada  France | In-depth semi-structured interviews | Cardiology, Inpatient wards (7), Outpatient (4), (Left Ventricular Assist Device (LVAD) vs. no LVAD) | *N*=30, HCP (surgeons n=1, cardiologists n=6, nurses n=3, social workers n=2, palliative care doctors n=1, physician assistants n=2)  Health service administrators (LVAD program coordinators n=11 - typically nurses or physician assistants, patient care service directors 1)  Government policy maker (Government program coordinator n=1)  Other stakeholders (industry market development managers n=2)  (HCP, HSA, HSDM, GPM, other stakeholders) | Systematically developed an 8-page LVAD pamphlet and 26-minute video Patient Decision Aid. Listed for free on the Ottawa Hospital Research Institute's patient decision aid page. Available in English and French |
| 14  (46) | van Veenendaal et al., (2018), The Netherlands | Focus Groups  In-depth semi-structured interviews  Written consultation feedback | Many *“participants were employed in primary and secondary healthcare (including mental healthcare), long-term care, patient advocacy, policy making (including health care insurance), management, research, or in companies that develop or implement decision aids.”* | *N*=113, n=32 health care professionals, n=10 patient advocates, n=41 researchers, n=25 Policy makers, n=5 other stakeholders  (Patient, HCP, HSA, GPM, other stakeholders) | SDM in general |
