# Supplementary material for: Barriers and facilitators to shared decision-making in hospitals from policy to practice: a systematic review
Source: Implement Sci. 2021 Jul 31;16:74. doi: 10.1186/s13012-021-01142-y (PMC8325317; doi:10.1186/s13012-021-01142-y)
Supplement: Supplementary file 3 — Additional File 3. Modified Critical Appraisal Skills Programme (CASP) Tool (table containing results of the CASP tool for each of the included studies). [file 13012_2021_1142_MOESM3_ESM.docx]

**Modified Critical Appraisal Skills Programme (CASP) Tool**

|  | | **Was there a clear statement of the aims of the research?** | **Is a qualitative methodology appropriate?** | **Was the research design appropriate to address the aims of the research?** | **Was the recruitment strategy appropriate to the aims of the research?** | **Was the data collected in a way that addressed the research issue?** | | **Has the relationship between researcher and participants been adequately considered?** | **Have ethical issues been taken into consideration?** | **Was the data analysis sufficiently rigorous?** | | **Is there a clear statement of findings?** |
| --- | --- | --- | --- | --- | --- | --- | --- | --- | --- | --- | --- | --- |
| Allen et al., (2020) (44) | Yes | | Yes | Yes | Somewhat^a^ | Yes | Yes | | Yes | Yes | Somewhat^b^ | |
| Barrett et al., (2016) (45) | | Yes | Yes | No^c^ | Can’t Tell^d^ | Can’t Tell^e^ | | Can’t Tell^f^ | Can’t Tell^g^ | No^h^ | | Somewhat^i^ |
| Chong et al., (2013) (50) | | Yes | Yes | Yes | Yes | Yes | | Yes | Yes | Yes | | Yes |
| Giacco et al., (2018) (51) | | Yes | Yes | Yes | Yes | Yes | | Can’t Tell^j^ | Yes | Yes | | Yes |
| Grant et al., (2020) (51) | | Yes | Yes | Yes | Yes | Yes | | Yes | Yes | Yes | | Yes |
| Hahlweg et al., (2016) (53) | | Yes | Yes | Yes | Yes | Yes | | Can’t Tell^k^ | Yes | Yes | | Yes |
| Hamann et al., (2016) (54) | | Yes | Yes | Yes | Yes | Yes | | Can’t Tell^l^ | Yes | Yes | | Yes |
| Pyl & Menard, (2012) (49) | | Yes | Yes | Somewhat^m^ | Yes | Somewhat^n^ | | Can’t Tell^o^ | Yes | No^p^ | | Somewhat^q^ |
| Schoenfeld et al., (2016) (55) | | Yes | Yes | Yes | Yes | Yes | | Yes | Yes | Yes | | Yes |
| Schoenfeld et al., (2018) (48) | | Yes | Yes | Yes | Yes | Yes | | Yes | Yes | Yes | | Yes |
| Schoenfeld, Goff, Downs, et al., (2018) (47) | | Yes | Yes | Yes | Yes | Yes | | Yes | Yes | Yes | | Yes |
| Schoenfeld et al., (2019) (56) | | Yes | Yes | Yes | Yes | Yes | | Yes | Yes | Yes | | Yes |
| Thompson et al., (2018) (57) | | Yes | Yes | Yes | Yes | Yes | | Yes | Yes | Yes | | Yes |
| van Veenendaal et al., (2018) (46) | | Yes | Yes | Yes | Yes | Yes | | Yes | Somewhat^r^ | Somewhat^s^ | | Yes |

Key

1. There is no explanation or discussion of recruitment (i.e. who chose to take part/not).
2. There are explicit findings, however it is not clear where these findings originate in the data and credibility is not discussed.
3. The researchers did not justify their research design, the methods used to obtain data are not clear.
4. The recruitment strategy and participant characteristics are not reported. There is no explanation of why participants were chosen, there is no explanation or discussion of recruitment (i.e. who chose to take part/not).
5. The data collection method is not reported, the setting and methods are not justified, the methods used for data collection are not explicit.
6. The researchers have not reported their own role and potential biases, the researchers have not reported how they responded to events during the study or any changes needed.
7. Ethical issues have not been reported. It is not clear whether ethics was obtained for this study.
8. There is no description of the analysis process, it is not clear how themes were derived from the data, data are not presented to support the findings.
9. There are explicit findings, however it is not clear where these findings originate in the data and credibility is not discussed.
10. The researchers have not reported their own role and potential biases, the researchers have not reported how they responded to events during the study or any changes needed.
11. The researchers have not reported their own role and potential biases, the researchers have not reported how they responded to events during the study or any changes needed, they have not reported the potential of the Hawthorne effect by using observation.
12. The researchers have not reported how they responded to events during the study or any changes needed.
13. The use of mixed methods research design is not justified.
14. Researchers have not made the method for obtaining qualitative data explicit.
15. The researchers have not reported their own role and potential biases, the researchers have not reported how they responded to events during the study or any changes needed, they have not reported the potential bias of the Hawthorne effect by using observation.
16. There is a limited explanation of the analysis process. It is not clear how the data were selected from the original sample, there is not sufficient data to support the findings.
17. The research findings are not explicit for barriers and facilitators.
18. Researchers report ethics approval was not require, however do not make explicit how the research was explained to participants or how confidentiality was handled.
19. Researchers do not explain how the presented data is selected from the original sample, there is not sufficient data presented to support the findings (i.e. no quotes are used).
