# Supplementary material for: Barriers and facilitators to shared decision-making in hospitals from policy to practice: a systematic review
Source: Implement Sci. 2021 Jul 31;16:74. doi: 10.1186/s13012-021-01142-y (PMC8325317; doi:10.1186/s13012-021-01142-y)
Supplement: Supplementary file 4 — Additional File 4. Barriers and Facilitators by frequency of citation to SDM Mapped to the TDF for Multiple Stakeholders (table containing frequency of citations of SDM mapped to the TDF where n=number of citations). [file 13012_2021_1142_MOESM4_ESM.docx]

**Barriers and Facilitators by frequency of citation to SDM Mapped to the TDF for Multiple Stakeholders, (n= number of citations)**

| **Domain** | **Clinician-related factors** | **Patient-related factors** | **Other stakeholder- related factors** | **Organisational-related factors** | **System-related factors** |
| --- | --- | --- | --- | --- | --- |
| 1. Knowledge   (An awareness of the existence of something) | n=5 | n=10 | n=1 | n=0 | n=1 |
|  | n=3 | n=8 | n=0 | n=3 | n=3 |
| 1. Skills   (An ability or proficiency acquired through practice) | n=6 | n=7 | n=0 | n=2 | n=4 |
|  | n=11 | n=7 | n=0 | n=4 | n=2 |
| 1. Social/Professional Role and Identity   (A coherent set of behaviours and displayed personal qualities of an individual in a social or work setting) | n=5 | n=5 | n=0 | n=2 | n=0 |
|  | n=10 | n=7 | n=2 | n=3 | n=1 |
| 1. Beliefs about Capabilities   (Acceptance of the truth, reality, or validity about an ability, talent, or facility that a person can put to constructive use) | n=5 | n=4 | n=0 | n=2 | n=2 |
|  | n=5 | n=5 | n=0 | n=0 | n=0 |
| 1. Optimism   (The confidence that things will happen for the best or that desired goals will be attained) | n=4 | n=1 | n=0 | n=0 | n=2 |
|  | n=0 | n=2 | n=0 | n=0 | n=0 |
| 1. Beliefs about Consequences   (Acceptance of the truth, reality, or validity about outcomes of a behaviour in a given situation) | n=6 | n=1 | n=1 | n=0 | n=0 |
|  | n=6 | n=0 | n=0 | n=1 | n=0 |
| 1. Reinforcement   (Increasing the probability of a response by arranging a dependent relationship, or contingency, between the response and a given stimulus) | n=3 | n=0 | n=0 | n=1 | n=2 |
|  | n=2 | n=0 | n=0 | n=0 | n=2 |
| 1. Intentions   (A conscious decision to perform a behaviour or a resolve to act in a certain way) | n=8 | n=2 | n=1 | n=3 | n=0 |
|  | n=3 | n=2 | n=0 | n=0 | n=0 |
| 1. Goals   (Mental representations of outcomes or end states that an individual wants to achieve) | n=0 | n=2 | n=0 | n=2 | n=0 |
|  | n=0 | n=0 | n=0 | n=0 | n=1 |
| 1. Memory, Attention and Decision Processes   (The ability to retain information, focus selectively on aspects of the environment and choose between two or more alternatives) | n=5 | n=3 | n=0 | n=2 | n=0 |
|  | n=2 | n=1 | n=0 | n=0 | n=0 |
| 1. Environmental Context and Resources   (Any circumstance of a person's situation or environment that discourages or encourages the development of skills and abilities, independence, social competence, and adaptive behaviour) | n=11 | n=10 | n=0 | n=6 | n=3 |
|  | n=3 | n=10 | n=2 | n=3 | n=4 |
| 1. Social Influences   (Those interpersonal processes that can cause individuals to change their thoughts, feelings, or behaviours) | n=6 | n=4 | n=3 | n=5 | n=1 |
|  | n=6 | n=0 | n=0 | n=6 | n=0 |
| 1. Emotion   (A complex reaction pattern, involving experiential, behavioural, and physiological elements, by which the individual attempts to deal with a personally significant matter or event) | n=3 | n=8 | n=0 | n=0 | n=0 |
|  | n=0 | n=2 | n=0 | n=0 | n=0 |
| 1. Behavioural Regulation   (Anything aimed at managing or changing objectively observed or measured actions) | n=0 | n=2 | n=0 | n=0 | n=0 |
|  | n=1 | n=4 | n=0 | n=4 | n=0 |
